# Interpregnancy Interval and Birth Outcomes: A Propensity Matching Study in the California Population

**DOI:** 10.1007/s10995-022-03388-4

**Published:** 2022-03-09

**Authors:** Jayme L. Congdon, Rebecca J. Baer, Jennet Arcara, Sky K. Feuer, Anu Manchikanti Gómez, Deborah Karasek, Scott P. Oltman, Matthew S. Pantell, Kelli Ryckman, Laura Jelliffe-Pawlowski

**Affiliations:** 1 Department of Pediatrics, University of California, San Francisco, 550 16th Street, San Francisco, CA 94158 USA; 2 Department of Pediatrics, University of California, San Diego, 9500 Gilman Drive, La Jolla, CA 92093 USA; 3 California Preterm Birth Initiative, University of California, San Francisco, 550 16th Street, San Francisco, CA 94158 USA; 4 Sexual Health and Reproductive Equity Program, School of Social Welfare, University of California, Berkeley, 120 Haviland Hall #7400, Berkeley, CA 94720-7400 USA; 5 Department of Obstetrics, Gynecology, and Reproductive Sciences, University of California, San Francisco, 550 16th Street, San Francisco, CA 94158 USA; 6 Departments of Epidemiology and Pediatrics, University of Iowa, 145 N. Riverside Drive, Iowa City, IA 52242 USA; 7 Department of Epidemiology and Biostatistics, University of California, San Francisco, 550 16th Street, San Francisco, CA 94158 USA

**Keywords:** Birth intervals, Family planning, Pregnancy outcome, Premature birth, Birth weight

## Abstract

**Introduction:**

Previous studies that used traditional multivariable and sibling matched analyses to investigate interpregnancy interval (IPI) and birth outcomes have reached mixed conclusions about a minimum recommended IPI, raising concerns about confounding. Our objective was to isolate the contribution of interpregnancy interval to the risk for adverse birth outcomes using propensity score matching.

**Methods:**

For this retrospective cohort study, data were drawn from a California Department of Health Care Access and Information database with linked vital records and hospital discharge records (2007–2012). We compared short IPIs of < 6, 6–11, and 12–17 months to a referent IPI of 18–23 months using 1:1 exact propensity score matching on 13 maternal sociodemographic and clinical factors. We used logistic regression to calculate the odds of preterm birth, early-term birth, and small for gestational age (SGA).

**Results:**

Of 144,733 women, 73.6% had IPIs < 18 months, 5.5% delivered preterm, 27.0% delivered early-term, and 6.0% had SGA infants. In the propensity matched sample (n = 83,788), odds of preterm birth were increased among women with IPI < 6 and 6–11 months (OR 1.89, 95% CI 1.71–2.0; OR 1.22, 95% CI 1.13–1.31, respectively) and not with IPI 12–17 months (OR 1.01, 95% CI 0.94–1.09); a similar pattern emerged for early-term birth. The odds of SGA were slightly elevated only for intervals < 6 months (OR 1.10, 95% CI 1.00–1.20, *p* < .05).

**Discussion:**

This study demonstrates a dose response association between short IPI and adverse birth outcomes, with no increased risk beyond 12 months. Findings suggest that longer IPI recommendations may be overly proscriptive.

**Supplementary Information:**

The online version contains supplementary material available at 10.1007/s10995-022-03388-4.

## Significance

### What is Already Known

Most clinical and public health recommendations define a short interpregnancy interval as < 18 months, though the evidence supporting this target has been called into question, citing concerns about confounding.

### What this Study Adds

Using propensity score matching to address confounding, we found a dose response association between interpregnancy interval and preterm birth. This study bolsters the evidence for a connection between interpregnancy intervals < 6 months and adverse birth outcomes and reveals a modest or null association for IPI durations beyond 6 months. These findings suggest that reconsideration of clinical and public health birth spacing targets may be warranted.

## Introduction

Short interpregnancy interval (IPI), commonly defined as < 18 months between a livebirth and subsequent conception (Gemmill & Lindberg, 2013), may increase the risk of adverse birth outcomes such as preterm birth, (Ahrens et al., 2018; Conde-Agudelo et al., 2006; DeFranco et al., 2014), early-term birth (DeFranco et al., 2014), or small for gestational age (SGA; Ahrens et al., 2018; Conde-Agudelo et al., 2006). Evidence of this association prompted the incorporation of birth spacing recommendations into U.S. clinical guidelines (American Academy of Family Physicians, 2015; Gavin et al., 2014; Louis et al., 2019) and public health objectives (US Department of Health and Human Services & Office of Disease Prevention & Health Promotion, 2010). Reducing short IPI has been considered a promising population health target given the high prevalence of short IPI (about 30% of U.S. pregnancies; Ahrens & Hutcheon, 2018), plausible causal mechanisms for the link between short IPI and adverse birth outcomes (Goldenberg et al., 2008), presumed modifiability of IPI, and potential for reducing health inequities (Appareddy et al., 2017). However, questions remain about the evidence base for IPI recommendations (Klebanoff, 2017). An expert working group convened by the U.S. Office of Population Affairs to synthesize IPI and birth outcomes research (Ahrens & Hutcheon, 2018) published an updated review focused on high resource settings, concluding that most, but not all, relevant studies support a link between IPI < 6 months and adverse perinatal outcomes (Ahrens et al., 2018). However, they highlight the uncertainty about IPI in the 6–18 month range and emphasize the need for research addressing some of the methodological limitations of published studies (Hutcheon, Moskosky, et al., 2018; Hutcheon, Nelson, et al., 2018). Importantly, prolonging IPI may not align with individual priorities influencing pregnancy spacing decisions (Callegari et al., 2017), and delaying pregnancy may lead to decreased fertility (Schmidt et al., 2012). These factors underscore the importance of clear evidence to guide public health initiatives, clinical counselling, and individual decision making.

Studies investigating IPI and birth outcomes have generally employed either multivariable regression or sibling comparison models (Hutcheon, Moskosky, et al., 2018; Hutcheon, Nelson, et al., 2018). However, these study designs may be vulnerable to bias from social factors and potential lack of generalizability (Hutcheon, Moskosky, et al., 2018; Hutcheon, Nelson, et al., 2018). Given our reliance upon observational data to elucidate the role of IPI in birth outcomes, propensity score matching may be an alternative method to reduce residual confounding from social factors. Approximating a controlled experiment, propensity score matching generates exposed and unexposed groups that are matched in terms of sociodemographic and other covariates that are hypothesized to relate to the outcome of interest (Austin, 2011). Two studies have employed these methods to investigate IPI and birth outcomes (Goyal et al., 2017; Howard et al., 2013). Of note, both treated short IPI as a dichotomous variable, which limits comparison to the majority of prior studies that analyzed intervals of < 6 months, 6–11 months, and 12–17 months relative to an 18–23 month reference interval (Hutcheon, Moskosky, et al., 2018; Hutcheon, Nelson, et al., 2018). Therefore, to isolate the contribution of short IPI to the risk of adverse birth outcomes in a manner that facilitates contextualization in the existing literature, we used these more granular IPI categories and a propensity score matching design in the California population. For comparison, we also conducted conventional multivariable logistic regression in an unmatched sample.

## Methods

### Cohort Selection

**Fig. 1:**
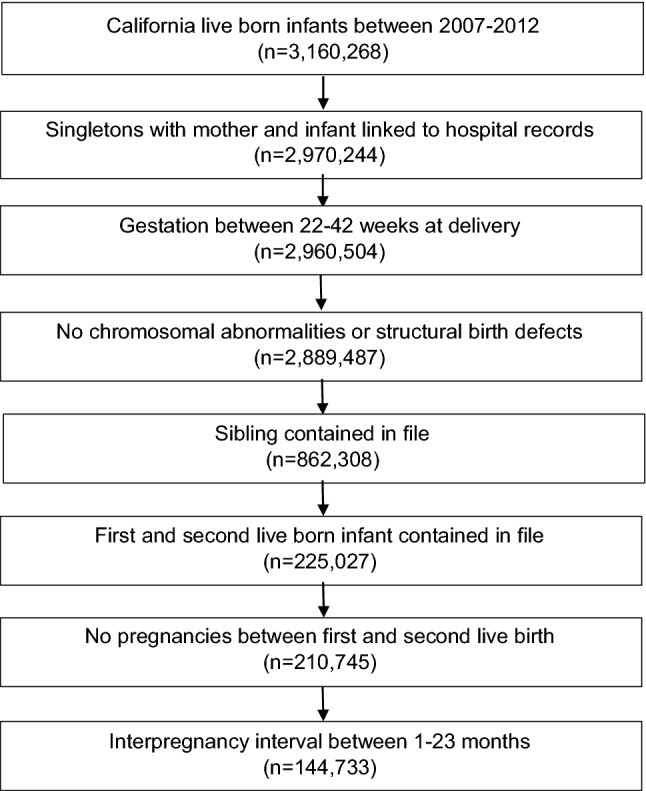
Sample selection

The sample for this retrospective cohort study was drawn from the Linked Birth Files database that is maintained by the California Department of Health Care Access and Information. The dataset includes linked maternal and infant vital statistics and hospital records for the nine months before and one year after delivery (Baer et al., 2014; Jelliffe-Pawlowski et al., 2015; Shachar et al., 2016; Yang et al., 2016). We included singleton births in California between 2007 and 2012 for whom vital records and hospital discharge records were linked and available in the dataset. Inclusion criteria were: gestational age at delivery between 22 and 42 weeks, infants with no chromosomal abnormalities or structural birth defects (Baer et al., 2014), first and second live birth for a woman contained in the data file, no interval pregnancies between the two live births, and an IPI between 1 and 23 months (Fig. 1). The study was conducted in accord with prevailing ethical principles, and the protocol was approved by the Committee for the Protection of Human Subjects within the Health and Human Services Agency of the State of California.

### Predictor Variables

IPI was measured as the time between the delivery of the first pregnancy to the expected last menstrual period of the second pregnancy. Expected last menstrual period was calculated by subtracting gestational age at birth of the second delivery from the birth date of the second infant. To facilitate comparison to most prior studies of short IPI and birth outcomes (Hutcheon, Moskosky, et al., 2018; Hutcheon, Nelson, et al., 2018), short IPI groups were designated as < 6 months, 6–11 months, 12–17 months, and the reference IPI was 18–23 months. Maternal characteristics were compared among IPI groups using chi-squared tests. All maternal characteristics were obtained from records for the first birth, which are the data that would available to clinicians and individuals considering birth spacing implications. Characteristics obtained from birth records were: maternal age at delivery (< 18 years, 18–34 years, > 34 years), maternal education (< 12 years, 12 years, > 12 years), Medicaid payment for delivery, enrollment in the Women, Infant, and Children (WIC) program, race and ethnicity (white, non-Hispanic, Hispanic, Black, Asian, other), pre-pregnancy body mass index (BMI; underweight < 18.5 kg/m^2^, normal 18.5–24.9 kg/m^2^, overweight 25.0–29.9 kg/m^2^, obese ≥ 30 kg/m^2^), and smoking during pregnancy. Previous preterm birth was determined by gestational age at birth of the first delivery, according to vital records. Characteristics obtained from hospital discharge records were: gestational diabetes, gestational hypertension, preeclampsia, drug or alcohol use during pregnancy, and mental health diagnosis (Online Resource 1).

### Outcome Variables

The outcomes examined were preterm birth (< 37 weeks of gestation), early-term birth (37–38 weeks of gestation), and small for gestational age (SGA). We obtained gestational age at birth (best obstetric estimate) and birthweight from vital records. Preterm birth was subgrouped into preterm prelabor rupture of membranes (PPROM), spontaneous labor with intact membranes, or provider-initiated preterm birth. As previously described (Jelliffe-Pawlowski et al., 2015), all preterm births with an indication of PPROM on the infant’s vital record or in hospital discharge records were included in the PPROM group. Women with no indication of PPROM but who had vital record or hospital discharge records with an indication of preterm labor or tocolytic medication were included in the spontaneous labor with intact membranes group. Preterm births without an indication of PPROM or spontaneous labor or tocolytic medication but with a code for medical induction, artificial rupture of membranes, or cesarean delivery were placed in the provider-initiated group. Preterm births were also subgrouped by gestational age: < 32 completed weeks or 32–36 completed weeks. SGA was defined as birthweight < 10th percentile for age and sex (Talge et al., 2014).

### Statistical Analysis

In the full, unmatched sample, we used logistic regression with a Poisson distribution to calculate unadjusted relative risks (RRs) and 95% confidence intervals (CIs) for preterm birth, early-term birth, SGA, PPROM, spontaneous preterm labor, and provider-initiated preterm birth for IPI < 6 months, 6–11 months, or 12–17 months, with a referent IPI of 18–23 months. We then calculated adjusted RRs for all maternal characteristics considered (Table 1).

**Table 1:** Maternal characteristics and obstetric factors among women with short interpregnancy interval and a reference interpregnancy interval (n = 144,733)

Maternal characteristics	Interpregnancy interval subgroups						
	< 6 months n = 16,903		6–11 months n = 40,985		12–17 months n = 48,710		18–23 months (Ref) n = 38,135
	n (%)	*p*	n (%)	*p*	n (%)	*p*	n (%)
Maternal age							
< 18 years	1286 (7.6)	< 0.001	1956 (4.8)	< 0.001	1970 (4.0)	0.265	1600 (4.2)
18–34 years	14,586 (86.3)	0.400	34,834 (85.0)	< 0.001	41,578 (85.4)	< 0.001	33,009 (86.6)
> 34 years	1031 (6.1)	< 0.01	4194 (10.2)	< 0.001	5161 (10.6)	< 0.001	3525 (9.2)
Maternal education							
< 12 years	3537 (20.9)	< 0.001	5207 (12.7)	< 0.001	4795 (9.8)	0.598	3795 (10.0)
12 years	5489 (32.5)	< 0.001	9797 (23.9)	< 0.001	9487 (19.5)	0.002	7743 (20.3)
> 12 years	7371 (43.6)	< 0.001	24,701 (60.3)	< 0.001	32,858 (67.5)	0.024	25,447 (66.7)
Medicaid payment for delivery	8723 (51.6)	< 0.001	13,858 (33.8)	< 0.001	12,916 (26.5)	0.910	10,125 (26.6)
Enrolled in WIC	9860 (58.3)	< 0.001	16,050 (39.2)	< 0.001	15,168 (31.1)	0.305	11,999 (31.5)
Race and ethnicity							
White, not hispanic	4258 (25.2)	< 0.001	15,211 (37.1)	< 0.001	20,736 (42.6)	0.134	16,041 (42.1)
Hispanic	8062 (47.7)	< 0.001	14,236 (34.7)	< 0.001	14,459 (29.7)	0.051	11,553 (30.3)
Black	1197 (7.1)	< 0.001	2119 (5.2)	< 0.001	1928 (4.0)	0.278	1565 (4.1)
Asian	1973 (11.7)	< 0.001	6355 (15.5)	0.0001	8134 (16.7)	0.477	6299 (16.5)
Other	1413 (8.4)	< 0.001	3064 (7.5)	0.014	3453 (7.1)	0.693	2677 (7.0)
Body mass index							
Underweight (< 18.5 kg/m2)	874 (5.2)	0.006	2216 (5.4)	0.033	2677 (5.5)	0.098	2195 (5.8)
Normal (18.5–24.9 kg/m2)	7852 (46.5)	< 0.001	22,214 (54.2)	< 0.001	27,950 (57.4)	0.883	21,863 (57.3)
Overweight (25.0–29.9 kg/m2)	3746 (22.2)	< 0.001	8340 (20.4)	< 0.001	9478 (19.5)	0.378	7331 (19.2)
Obese (≥ 30 kg/m2)	3325 (19.7)	< 0.001	5798 (14.2)	< 0.001	5880 (12.1)	0.483	4544 (11.9)
Gestational Diabetes	1062 (6.3)	0.093	2549 (6.2)	0.072	2775 (5.7)	0.176	2255 (5.9)
Gestational Hypertension	739 (4.4)	0.573	1777 (4.3)	0.631	1966 (4.0)	0.091	1627 (4.3)
Preeclampsia	912 (5.4)	< 0.001	1826 (4.5)	0.148	2023 (4.2)	0.501	1619 (4.3)
Smoking during Pregnancy	914 (5.6)	< 0.001	1591 (3.9)	< 0.001	1511 (3.10	0.131	1252 (3.3)
Drug or Alcohol Abuse	426 (2.5)	< 0.001	701 (1.7)	< 0.001	602 (1.2)	0.080	422 (1.1)
Mental Health Diagnosis	572 (3.4)	< 0.001	1097 (2.7)	< 0.001	1069 (2.2)	0.638	855 (2.2)
Previous Preterm Birth	1462 (8.7)	< 0.001	2776 (6.8)	0.0006	2943 (6.0)	0.415	2355 (6.2)

*WIC* women, infants, and children program

We used logistic regression to create propensity scores for IPI < 6 months, 6–11 months, or 12–17 months for all maternal characteristics (Table 1). A referent population of women with an IPI of 18–23 months was randomly selected at a 1:1 ratio with women in the < 6 month, 6–11 month, and 12–17 month IPI groups, using exact matching of propensity scores without replacement. Although we selected propensity-matched controls within an IPI group without replacement, the entire population of women with an IPI between 18 and 23 months was available for each IPI group being analyzed. Women without an exact propensity score-matched control were not included in the analyses. Characteristics and outcomes for women with an exact match were compared to women without an exact match using chi squared tests. Logistic regression was used to calculate odds ratios (OR) and 95% CIs for preterm birth, early-term birth, SGA, PPROM, spontaneous preterm labor, and provider-initiated preterm birth for propensity score-matched women with an IPI < 6 months, 6–11 months, or 12–17 months. All analyses were performed using Statistical Analysis Software version 9.4 (Cary, NC).

## Results

### Sample Description

The full, unmatched sample included 144,733 women, of whom 12% had an IPI < 6 months, 28% had an IPI between 6 and 11 months, 34% had an IPI between 12 and 17 months, and 26% had an IPI between 18 and 23 months (Table 1). Maternal characteristics differed for women with an IPI < 6 months or 6–11 months versus the referent population of women with an IPI of 18–23 months. Demographically, a greater proportion of women with short IPIs were < 18 years of age, of Black race or Hispanic ethnicity, enrolled in WIC or Medicaid, or had completed fewer years of education at the first birth. The shortest IPI groups also included more women with a prior preterm birth and medical comorbidities (e.g., preeclampsia, smoking, mental health diagnosis). Women with an IPI between 12 and 17 months were generally similar to the referent population.

### Regression Analyses in the Unmatched Sample

**Fig. 2:**
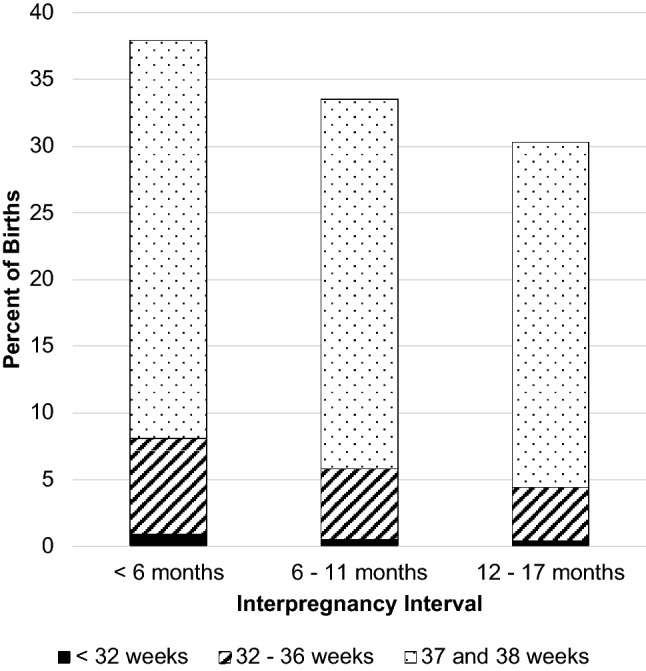
Rate of Preterm and Early Term Birth by Interpregnancy Interval (n = 144,733)

In the full, unmatched sample, the proportion of women with a preterm birth declined with progressively longer IPI, ranging from 8.7% of women with an IPI < 6 months to 4.7% of women with an IPI between 18 and 23 months (Table 2, Fig. 2). Women with an IPI of less than 6 months were at increased risk of a preterm (< 37 weeks) or early-term (37–38 weeks) birth (aRR 1.70, 95% CI 1.58, 1.83, aRR 1.17, 95% CI 1.13, 1.21). Women with an IPI between 6 and 11 months also had a statistically significant increased risk of a preterm or early-term birth, although the association was not marked. Women with an IPI between 12 and 17 months were not at elevated risk of a preterm or early-term birth in unadjusted or adjusted models when compared to women with an IPI between 18 and 23 months.

**Table 2:** Risk of preterm birth, early term birth, and small for gestational age by interpregnancy interval in full, unmatched sample (n = 144,733)

Birth outcome	Interpregnancy interval subgroups n (%) RR (95% CI) aRR (95% CI)^a^			
	< 6 months n = 16,903	6–11 months n = 40,985	12–17 months n = 48,710	18–23 months n = 38,135
< 32 weeks GA	173 (1.0)	225 (0.6)	218 (0.5)	145 (0.4)
	3.03 (2.43, 3.78)	1.50 (1.22, 1.85)	1.19 (0.96, 1.46)	Ref
	2.38 (1.88, 3.00)	1.37 (1.11, 1.69)	1.19 (0.96, 1.46)	Ref
32–36 weeks GA	1292 (7.6)	2111 (5.2)	2109 (4.3)	1632 (4.3)
	1.92 (1.78, 2.06)	1.24 (1.16, 1.32)	1.02 (0.96, 1.09)	Ref
	1.67 (1.54, 1.80)	1.18 (1.11, 1.26)	1.02 (0.95, 1.09)	Ref
< 37 weeks GA (any preterm)	1465 (8.7)	2336 (5.7)	2327 (4.8)	1777 (4.7)
	1.98 (1.85, 2.12)	1.26 (1.18, 1.34)	1.03 (0.97, 1.10)	Ref
	1.70 (1.58, 1.83)	1.19 (1.12, 1.27)	1.03 (0.97, 1.10)	Ref
37–38 weeks GA (early term)	5106 (30.2)	11,349 (27.7)	12,750 (26.2)	9810 (25.7)
	1.23 (1.19, 1.27)	1.09 (1.06, 1.12)	1.01 (0.99, 1.05)	Ref
	1.17 (1.13, 1.21)	1.07 (1.04, 1.10)	1.01 (0.99, 1.05)	Ref
SGA	1234 (7.3)	2494 (6.1)	2735 (5.6)	2226 (5.8)
	1.25 (1.17, 1.34)	1.04 (0.98, 1.10)	0.96 (0.91, 1.02)	Ref
	1.10 (1.02, 1.19)	1.00 (0.95, 1.06)	0.97 (0.91, 1.02)	Ref

*aRR* adjusted relative risk, *CI* confidence interval, *GA* gestational age at birth, *RR* relative risk, *SGA* small for gestational age

^a^Risks adjusted for maternal characteristics in Table 1

Women with an IPI < 6 months were at slightly increased risk of an SGA infant (aRR 1.10, 95% CI 1.02, 1.19). Women with an IPI between 6 and 11 months or 12–17 months did not have a significantly increased risk of having an SGA infant for compared to women with an IPI of 18–23 months (Table 2).

### Regression Analyses in the Propensity-Matched Sample

Exact propensity score matches were made for 87.3% of women with an IPI < 6 months, 72.4% with an IPI between 6 and 11 months, and 74.1% with an IPI between 12 and 17 months (Table 3). Women with an exact match, who were included in the propensity-matched sample, differed from unmatched women in terms of age distribution, race and ethnicity, socioeconomic status, and prenatal comorbidities (Online Resource 2).

**Table 3:** Maternal characteristics by interpregnancy interval in a propensity score-matched sample (n = 83,788)

Maternal characteristics	Interpregnancy interval subgroups n (%)					
	< 6 months n = 14,748	18–23 months^a^ n = 14,748	6–11 months n = 29,668	18–23 months^a^ n = 29,668	12–17 months n = 36,108	18–23 months^a^ n = 36,108
Maternal age						
< 18 years	1017 (6.9)	1017 (6.9)	1269 (4.3)	1269 (4.3)	1306 (3.6)	1306 (3.6)
18–34 years	12,906 (87.5)	12,906 (87.5)	25,257 (85.1)	25,257 (85.1)	31,550 (87.4)	31,550 (87.4)
> 34 years	825 (5.6)	825 (5.6)	3141 (10.6)	3141 (10.6)	3251 (9.0)	3251 (9.0)
Maternal education						
< 12 years	2760 (18.7)	2760 (18.7)	3238 (10.9)	3238 (10.9)	3255 (9.0)	3255 (9.0)
12 years	4789 (32.5)	4789 (32.5)	7065 (23.8)	7065 (23.8)	7011 (19.4)	7011 (19.4)
> 12 years	6859 (46.5)	6859 (46.5)	18,510 (62.4)	18,510 (62.4)	24,714 (68.4)	24,714 (68.4)
Medicaid payment for delivery	7270 (49.3)	7270 (49.3)	9120 (30.7)	9120 (30.7)	9175 (25.4)	9175 (25.4)
Enrolled in WIC	8379 (56.8)	8379 (56.8)	10,875 (36.7)	10,875 (36.7)	10,929 (30.3)	10,929 (30.3)
Race and ethnicity						
White, not hispanic	3819 (25.9)	3819 (25.9)	10,583 (35.7)	10,583 (35.7)	15,441 (42.8)	15,441 (42.8)
Hispanic	7233 (49.0)	7233 (49.0)	10,767 (36.3)	10,767 (36.3)	10,942 (30.3)	10,942 (30.3)
Black	851 (5.8)	851 (5.8)	1279 (4.3)	1279 (4.3)	1287 (3.6)	1287 (3.6)
Asian	1745 (11.8)	1745 (11.8)	4869 (16.4)	4869 (16.4)	6054 (16.8)	6054 (16.8)
Other	1100 (7.5)	1100 (7.5)	2170 (7.3)	2170 (7.3)	2384 (6.6)	2384 (6.6)
Body mass index						
Underweight (< 18.5 kg/m^2^)	743 (5.0)	743 (5.0)	1708 (5.8)	1708 (5.8)	1924 (5.3)	1924 (5.3)
Normal (18.5–24.9 kg/m^2^)	7281 (49.4)	7281 (49.4)	15,776 (53.2)	15,776 (53.2)	21,319 (59.0)	21,319 (59.0)
Overweight (25.0–29.9 kg/m^2^)	3269 (22.2)	3269 (22.2)	6482 (21.9)	6482 (21.9)	6824 (18.9)	6824 (18.9)
Obese (≥ 30 kg/m^2^)	2626 (17.8)	2626 (17.8)	3971 (13.4)	3971 (13.4)	4068 (11.3)	4068 (11.3)
Gestational diabetes	697 (4.7)	697 (4.7)	1686 (5.7)	1686 (5.7)	1832 (5.1)	1832 (5.1)
Gestational Hypertension	483 (3.3)	483 (3.3)	1133 (3.8)	1133 (3.8)	1261 (3.5)	1261 (3.5)
Preeclampsia	562 (3.8)	562 (3.8)	1017 (3.4)	1017 (3.4)	1219 (3.4)	1219 (3.4)
Smoking during Pregnancy	482 (3.3)	482 (3.3)	779 (2.6)	779 (2.6)	837 (2.3)	837 (2.3)
Drug or alcohol abuse	115 (0.8)	115 (0.8)	171 (0.6)	171 (0.6)	182 (0.5)	182 (0.5)
Mental health diagnosis	185 (1.3)	185 (1.3)	422 (1.4)	422 (1.4)	476 (1.3)	476 (1.3)
Previous preterm birth	848 (5.8)	848 (5.8)	1624 (5.5)	1624 (5.5)	1810 (5.0)	1810 (5.0)

*WIC* women, infants, and children program

^a^Reference interpregnancy interval

**Fig. 3:**
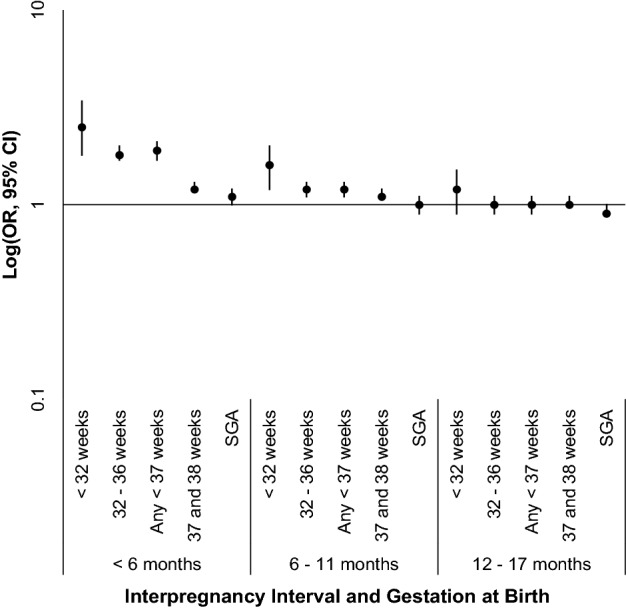
Odds of Preterm or Early Term Birth by Interpregnancy Interval in a Propensity Score-Matched Sample (n = 83,788). *CI* confidence interval, *OR* odds ratio, *SGA* small for gestational age

In the propensity score-matched sample, findings were consistent with the unmatched regression model, demonstrating a significantly greater risk of preterm or early term birth among women with an IPI < 6 months, and a significant but less marked difference for women with an IPI between 6 and 11 months. Specifically, compared to women with an IPI between 18 and 23 months, women with an IPI < 6 months had higher odds of a preterm or early-term birth (OR 1.89, 95% CI 1.71, 2.08, OR 1.25, 95% CI 1.19, 1.31, respectively) (Table 4, Fig. 3). With an IPI < 6 months, the odds were especially elevated for birth < 32 weeks with spontaneous labor and intact membranes (OR 3.65, 95% CI 2.37, 5.62) (Online Resource 3). For women with IPIs of 6–11 months, the ORs were 1.22 for preterm birth, 1.11 for early term birth, and 1.82 for birth < 32 weeks with spontaneous labor and intact membranes (95% CIs 1.13, 1.31; 1.07, 1.15; and 1.30, 2.55; respectively). Women with an IPI between 12 and 17 months were not at increased odds of a preterm or early-term birth compared to women with an IPI of 18–23 months.

**Table 4:** Odds of preterm birth, early term birth, and small for gestational age by interpregnancy interval in a propensity score-matched sample (n = 83,788)

Birth outcome	Interpregnancy interval subgroups n (%) OR (95% CI)					
	< 6 months n = 14,748	18–23 months n = 14,748	6–11 months n = 29,668	18–23 months n = 29,668	12–17 months n = 36,108	18–23 months n = 36,108
< 32 weeks GA	131 (0.9)	58 (0.4)	155 (0.5)	102 (0.3)	142 (0.4)	120 (0.3)
	2.50 (1.83, 3.41)	Ref	1.58 (1.23, 2.03)	Ref	1.19 (0.94, 1.52)	Ref
32–36 weeks GA	1055 (7.2)	637 (4.3)	1427 (4.8)	1247 (4.2)	1455 (4.0)	1474 (4.1)
	1.83 (1.66, 2.03)	Ref	1.19 (1.10, 1.29)	Ref	1.00 (0.92, 1.07)	Ref
< 37 weeks GA (any preterm)	1186 (8.0)	695 (4.7)	1582 (5.3)	1349 (4.6)	1597 (4.4)	1594 (4.4)
	1.89 (1.71, 2.08)	Ref	1.22 (1.13, 1.31)	Ref	1.01 (0.94, 1.09)	Ref
37–38 weeks GA (early term)	4401 (29.8)	3907 (26.5)	8216 (27.7)	7691 (25.9)	9359 (25.9)	9156 (25.4)
	1.25 (1.19, 1.31)	Ref	1.11 (1.07, 1.15)	Ref	1.03 (1.00, 1.07)	Ref
SGA	1,024 (6.9)	938 (6.4)	1,753 (5.9)	1,763 (5.9)	1,949 (5.4)	2,069 (5.7)
	1.10 (1.00, 1.20)^a^	Ref	0.99 (0.93, 1.06)	Ref	0.94 (0.88, 1.00)	Ref

*GA* gestational age at birth, *SGA* small for gestational age, *OR* odds ratio

^a^*p* < 0.05

Women with an IPI < 6 months had slightly increased odds of an SGA infant (OR 1.10, 95% CI 1.00, 1.20, *p* < 0.05). Women with an IPI between 6 and 11 or 12–17 months were not at increased odds of an SGA infant (Table 4, Fig. 3).

## Discussion

In this population-based study, we observed a dose response relation between IPI duration and preterm or early-term birth. Our findings demonstrated the highest odds of preterm birth for an IPI < 6 months, a smaller but still elevated odds for an IPI of 6–11 months, and no increased odds for an IPI of 12–17 months. The odds of SGA were slightly elevated only among women with the shortest IPI of < 6 months. The results of our analyses using the full, unmatched sample aligned closely with the results from the propensity-matched sample, both in terms of magnitude of risk and level of confidence.

Our finding that the shortest IPI lengths conferred the greatest risk of adverse birth outcomes is consistent with the majority of prior studies that utilized multivariable regression or sibling matched methods (Ahrens et al., 2018; DeFranco et al., 2014) and with two prior studies that utilized propensity score analyses (Goyal et al., 2017; Howard et al., 2013). Previous conventional multivariable regression and sibling comparison studies analyzing IPIs of 6–11 and 12–17 months have yielded mixed results, with generally smaller point estimates and confidence intervals that included the null (Ahrens et al., 2018).

An important point of contrast is a previously published California population study that compared within-woman (sibling matching) to between-women (conventional multivariable) analyses; after adjusting for eight covariates, Shachar et al. found a dose response pattern of increased risk for preterm birth after short IPIs of up to 18 months (2016). In the present study, using a propensity score matching model with 13 social and clinical covariates, we found that the risk of preterm birth was no longer elevated beyond 12 months. Both studies used a reference interval of 18–23 months. The lack of an association between IPI of 12–17 months and preterm birth in the present study may be due to greater control of social and clinical cofounders. Another possibility is that the findings from the sibling matching study do not generalize to the broader population of reproductive age women. Sibling matching relies upon a sample of women with at least three live births with distinct IPI durations and different birth outcomes of the second and third pregnancies (Hutcheon, Moskosky, et al., 2018; Hutcheon, Nelson, et al., 2018).

Providing additional useful context, Howard et al. similarly compared the results from conventional multivariable regression to a propensity matched model. The models produced parallel findings, which was also true of the present study. The study by Howard et al. used different IPI categories (< 9 months versus 9–24 months), which limits comparison to the present study in further detail.

Taken together with other studies in high resource settings, our findings suggest that, for the purpose of optimizing birth outcomes, U.S. clinical guidelines and public health objectives may be overly conservative in recommending an IPI of > 18 months. The most recent clinical guidelines published in 2019 by the American College of Obstetricians and Gynecologists and Society for Maternal–Fetal Medicine offer a more nuanced take on birth spacing, specifying a strong (grade 1B) recommendation to prolong the IPI to > 6 months, alongside a weak (grade 2B) recommendation for an IPI of > 18 months (Louis et al., 2019). Conservative recommendations may clinically benefit individuals who are most susceptible to the adverse impact of short IPI on birth outcomes. However, overly conservative guidance has the potential to impede patient autonomy and engender mistrust in health services. As the empirical basis for birth spacing guidelines evolves, along with the growing literature on maternal outcomes after short IPI (Hutcheon, Moskosky, et al., 2018; Hutcheon, Nelson, et al., 2018), clinical care and public health objectives must adjust accordingly.

Given the complex and personal nature of family planning decision-making, less proscriptive medical recommendations could foster more individualized discussions and aid shared decision making between providers, women, and partners based upon personal circumstances and goals (Callegari et al., 2017). Such a shift would align with calls for family planning metrics that target quality of care (e.g. patient satisfaction, access), rather than metrics that incentivize the provision and uptake of the most effective contraceptive methods, an approach that may hinder patient autonomy and equity and negatively impact long-term health care seeking and health outcomes (Dehlendorf et al., 2015; Gomez et al., 2014, 2018).

### Strengths and Limitations

Our study has several strengths. First, the California population dataset we utilized contains a large, socioeconomically, racially, and ethnically diverse sample. The large sample size permitted utilization of a propensity matching design, which is a statistically robust means of using observational data to simulate a controlled experiment and isolate the predictor of interest (Austin, 2011). Additionally, the dataset included linked vital and hospital records, allowing for the inclusion of demographic, social, and clinical variables in the statistical model.

Our findings are most generalizable to women in the U.S. or other high resource regions. Important caveats are that the California preterm birth rate is lower than in other parts of the U.S. (Martin et al., 2019), though with worse racial and ethnic disparities than most U.S. states (March of Dimes, 2018). Also of note is our use of social and obstetric characteristics from the first pregnancy, limiting our ability to capture potential changes in these variables between the first and second pregnancies that may contribute to the outcomes of the second pregnancy. However, data from the first pregnancy are the data available to providers and individuals considering the timing of a subsequent pregnancy and thus have greater utility in clinical decision making. In addition, our sample was restricted to women with two live births and no intervening pregnancy losses or terminations, limiting inference about the risk associated with short IPI beyond a second live birth or for women with interval pregnancies.

A limitation of our propensity-matched analyses lies in the significant differences in maternal characteristics between women in the matched sample and women without a match. However, the closely aligned results from analyses using the full sample versus the propensity-matched sample offer reassurance that the exclusion of unmatched women from the propensity analyses did not overly affect our results.

Finally, similar to all prior studies of IPI and birth outcomes, ours too was limited by being based on observational data available in vital records and hospital databases. One implication of using this type of data is the limited number and specificity of social variables, lacking important factors with relevance to birth outcomes, such as racial discrimination (Chambers et al., 2019; Rosenberg et al., 2002), housing instability (Pantell et al., 2019), neighborhood context (Ncube et al., 2016), and pregnancy intendedness or wantedness (Shah et al., 2011). Using propensity score analyses, we assume that as exposure groups are matched in terms of measured social and demographic variables, some of the closely aligned unmeasured factors will be balanced as well, though additional residual confounding may have occurred. Of note, the propensity-matching design precludes subgroup analysis and thus does not permit investigation into the role of IPI in birth outcome disparities, which is a critical area of ongoing and future birth outcomes research.

### Future Directions

Propensity matching studies using data from other populations constitute an important future direction to assess the generalizability of our findings of: (1) a clear dose response association between IPI and preterm birth and (2) the similar findings from conventional multivariable versus propensity score-matched regression models. To address the limited availability of social covariates in birth and hospital records, richer databases comprised of survey data for representative samples may be useful in unpacking associations among detailed social and structural factors, pregnancy intention and acceptability, IPI, and birth outcomes. Future research should additionally assess the impact of changes in clinical guidelines or public health initiatives aimed at reducing short IPI to evaluate their impact on pregnancy spacing and birth outcomes. Research investigating methods for incorporating IPI evidence into patient-centered approaches to family planning discussions with women (Callegari et al., 2017) is an additional future area of inquiry.

## Conclusion

In this California population sample, compared to women with an IPI of 18–23 months, women with the shortest IPI of < 6 months had the highest risk of adverse birth outcomes, and women with an IPI of 12–17 months had no increased risk. These findings prompt re-evaluation of clinical guidelines and public health initiatives intended to improve health outcomes by promoting a minimum IPI of > 18 months.

## Supplementary Information

Below is the link to the electronic supplementary material.

Supplementary file1 (DOCX 20 kb)

## Data Availability

Data are available upon request to the California Department of Health Care Access and Information.

## Code Availability

We utilized standard statistical software (Statistical Analysis Software version 9.4).

## References

[CR1] Ahrens KA, Hutcheon JA (2018). Birth spacing in the United States—Towards evidence-based recommendations. Paediatric and Perinatal Epidemiology.

[CR2] Ahrens KA, Nelson H, Stidd RL, Moskosky S, Hutcheon JA (2018). Short interpregnancy intervals and adverse perinatal outcomes in high-resource settings: An updated systematic review. Paediatric and Perinatal Epidemiology.

[CR3] American Academy of Family Physicians. (2015). *Preconception Care (Position Paper)*. https://www.aafp.org/about/policies/all/preconception-care.html

[CR4] Appareddy S, Pryor J, Bailey B (2017). Inter-pregnancy interval and adverse outcomes: Evidence for an additional risk in health disparate populations. The Journal of Maternal-Fetal & Neonatal Medicine.

[CR5] Austin PC (2011). An introduction to propensity score methods for reducing the effects of confounding in observational studies. Multivariate Behavioral Research.

[CR6] Baer RJ, Norton ME, Shaw GM, Flessel MC, Goldman S, Currier RJ, Jelliffe-Pawlowski LL (2014). Risk of selected structural abnormalities in infants after increased nuchal translucency measurement. American Journal of Obstetrics and Gynecology.

[CR7] Callegari LS, Aiken ARA, Dehlendorf C, Cason P, Borrero S (2017). Addressing potential pitfalls of reproductive life planning with patient-centered counseling. American Journal of Obstetrics & Gynecology.

[CR8] Chambers BD, Baer RJ, McLemore MR, Jelliffe-Pawlowski LL (2019). Using index of concentration at the extremes as indicators of structural racism to evaluate the association with preterm birth and infant Mortality-California, 2011–2012. Journal of Urban Health: Bulletin of the New York Academy of Medicine.

[CR9] Conde-Agudelo A, Rosas-Bermúdez A, Kafury-Goeta AC (2006). Birth spacing and risk of adverse perinatal outcomes: A meta-analysis. JAMA.

[CR10] DeFranco EA, Ehrlich S, Muglia LJ (2014). Influence of interpregnancy interval on birth timing. BJOG an International Journal of Obstetrics & Gynaecology.

[CR11] Dehlendorf C, Bellanca H, Policar M (2015). Performance measures for contraceptive care: What are we actually trying to measure?. Contraception.

[CR12] Gavin, L., Moskosky, S., Carter, M., Curtis, K., Glass, E., Godfrey, E., Marcell, A., Mautone-Smith, N., Pazol, K., Tepper, N., Zapata, L., & Centers for Disease Control and Prevention (CDC). (2014). Providing quality family planning services: Recommendations of CDC and the U.S. Office of Population Affairs. *MMWR. Recommendations and Reports: Morbidity and Mortality Weekly Report. Recommendations and Reports*, *63*(RR-04), 1–54. 24759690

[CR13] Gemmill A, Lindberg LD (2013). Short interpregnancy intervals in the United States. Obstetrics and Gynecology.

[CR14] Goldenberg RL, Culhane JF, Iams JD, Romero R (2008). Epidemiology and causes of preterm birth. The Lancet.

[CR15] Gomez AM, Arteaga S, Ingraham N, Arcara J, Villaseñor E (2018). It’s not planned, but is it okay? The acceptability of unplanned pregnancy among young people. Women’s Health Issues.

[CR16] Gomez AM, Fuentes L, Allina A (2014). Women or LARC first? reproductive autonomy and the promotion of long-acting reversible contraceptive methods. Perspectives on Sexual and Reproductive Health.

[CR17] Goyal NK, Folger AT, Hall ES, Greenberg JM, Van Ginkel JB, Ammerman RT (2017). Home visiting for first-time mothers and subsequent pregnancy spacing. Journal of Perinatology.

[CR18] Howard EJ, Harville E, Kissinger P, Xiong X (2013). The association between short interpregnancy interval and preterm birth in Louisiana: A comparison of methods. Maternal and Child Health Journal.

[CR19] Hutcheon JA, Moskosky S, Ananth CV, Basso O, Briss PA, Ferré CD, Frederiksen BN, Harper S, Hernández-Díaz S, Hirai AH, Kirby RS, Klebanoff MA, Lindberg L, Mumford SL, Nelson HD, Platt RW, Rossen LM, Stuebe AM, Thoma ME, Ahrens KA (2018). Good practices for the design, analysis, and interpretation of observational studies on birth spacing and perinatal health outcomes. Paediatric and Perinatal Epidemiology.

[CR20] Hutcheon JA, Nelson HD, Stidd R, Moskosky S, Ahrens KA (2018). Short interpregnancy intervals and adverse maternal outcomes in high-resource settings: An updated systematic review. Paediatric and Perinatal Epidemiology.

[CR21] Jelliffe-Pawlowski LL, Baer RJ, Blumenfeld YJ, Ryckman KK, O’Brodovich HM, Gould JB, Druzin ML, El-Sayed YY, Lyell DJ, Stevenson DK, Shaw GM, Currier RJ (2015). Maternal characteristics and mid-pregnancy serum biomarkers as risk factors for subtypes of preterm birth. BJOG an International Journal of Obstetrics and Gynaecology.

[CR22] Klebanoff MA (2017). Interpregnancy interval and pregnancy outcomes: Causal or not?. Obstetrics & Gynecology.

[CR23] Louis JM, Bryant A, Ramos D, Stuebe A, Blackwell SC (2019). Interpregnancy care. American Journal of Obstetrics & Gynecology.

[CR24] March of Dimes. (2018). *Premature Birth Report Card*. https://www.marchofdimes.org/mission/prematurity-reportcard.aspx

[CR25] Martin, JA, Hamilton, BE, Osterman, MJK, & Driscoll, AK. (2019). *National Vital Statistics Reports: Births: Final data for 2018. 68*(13), 47. 32501202

[CR26] Ncube CN, Enquobahrie DA, Albert SM, Herrick AL, Burke JG (2016). Association of neighborhood context with offspring risk of preterm birth and low birthweight: A systematic review and meta-analysis of population-based studies. Social Science & Medicine.

[CR27] Pantell MS, Baer RJ, Torres JM, Felder JN, Gomez AM, Chambers BD, Dunn J, Parikh NI, Pacheco-Werner T, Rogers EE, Feuer SK, Ryckman KK, Novak NL, Tabb KM, Fuchs J, Rand L, Jelliffe-Pawlowski LL (2019). Associations between unstable housing, obstetric outcomes, and perinatal health care utilization. American Journal of Obstetrics & Gynecology MFM.

[CR28] Rosenberg L, Palmer JR, Wise LA, Horton NJ, Corwin MJ (2002). Perceptions of racial discrimination and the risk of preterm birth. Epidemiology.

[CR29] Schmidt L, Sobotka T, Bentzen JG, Nyboe Andersen A (2012). Demographic and medical consequences of the postponement of parenthood. Human Reproduction Update.

[CR30] Shachar BZ, Mayo JA, Lyell DJ, Baer RJ, Jeliffe-Pawlowski LL, Stevenson DK, Shaw GM (2016). Interpregnancy interval after live birth or pregnancy termination and estimated risk of preterm birth: A retrospective cohort study. BJOG an International Journal of Obstetrics & Gynaecology.

[CR31] Shah PS, Balkhair T, Ohlsson A, Beyene J, Scott F, Frick C (2011). Intention to become pregnant and low birth weight and preterm birth: A systematic review. Maternal and Child Health Journal.

[CR32] Talge NM, Mudd LM, Sikorskii A, Basso O (2014). United States birth weight reference corrected for implausible gestational age estimates. Pediatrics.

[CR33] US Department of Health and Human Services, & Office of Disease Prevention and Health Promotion. (2010). *Healthy People 2020*. https://www.healthypeople.gov/2020/topics-objectives/topic/family-planning

[CR34] Yang J, Baer RJ, Berghella V, Chambers C, Chung P, Coker T, Currier RJ, Druzin ML, Kuppermann M, Muglia LJ, Norton ME, Rand L, Ryckman K, Shaw GM, Stevenson D, Jelliffe-Pawlowski LL (2016). Recurrence of preterm birth and early term birth. Obstetrics and Gynecology.

