# Supplementary material for: Interpregnancy Interval and Birth Outcomes: A Propensity Matching Study in the California Population
Source: Matern Child Health J. 2022 Mar 9;26(5):1115–25. doi: 10.1007/s10995-022-03388-4 (PMC9023393; doi:10.1007/s10995-022-03388-4)
Supplement: Supplementary file 1 — Supplementary file1 (DOCX 20 kb) [file 10995_2022_3388_MOESM1_ESM.docx]

Electronic Supplementary Material

*Online Resource 1****.*** *Diagnostic Codes Used from Hospital Discharge Records*

| **Diagnosis** | **ICD-9 diagnostic code** |
| --- | --- |
| Drug or Alcohol Dependence/Abuse | 648.3 Drug dependence  305 Nondependent abuse of drugs  304 Drug dependence  303 Alcohol dependence syndrome |
| Gestational Diabetes | 648.8 Abnormal glucose tolerance |
| Gestational Hypertension | 642.3 Transient hypertension of pregnancy |
| Mental Disorder | 648.4 Mental disorders |
| Preeclampsia | 642.4 Mild or unspecified pre-eclampsia  642.5 Severe pre-eclampsia  642.6 Eclampsia  642.7 Pre-eclampsia or eclampsia superimposed on pre-existing hypertension |

*Online Resource 2. Comparison of Characteristics between Propensity Score- Matched Sample (n=83,788) and Unmatched Sample (n=26,074) by Interpregnancy Interval*

| **Maternal Characteristics** | **Interpregnancy Interval Subgroup** | | | | | | | | |
| --- | --- | --- | --- | --- | --- | --- | --- | --- | --- |
|  | <6 months | | | 6-11 months | | | 12-17 months | | |
|  | Matched  n=14,748 | Unmatched  n=2,155 |  | Matched  n=29,668 | Unmatched  n=11,317 |  | Matched  n=36,108 | Unmatched  n=2,602 |  |
|  | n (%) | n (%) | *p* | n (%) | n (%) | *p* | n (%) | n (%) | *p* |
| Maternal Age |  |  |  |  |  |  |  |  |  |
| <18 years | 1,017 (6.9) | 269 (12.5) | <.001 | 1,269 (4.3) | 687 (6.1) | <.001 | 1,306 (3.6) | 664 (5.3) | <.001 |
| 18-34 years | 12,906 (87.5) | 1,680 (78.0) | <.001 | 25,257 (85.1) | 9,577 (84.6) | .199 | 3,1,550 (87.4) | 10,028 (79.6) | <.001 |
| >34 years | 825 (5.6) | 206 (9.6) | <.001 | 3,141 (10.6) | 1,053 (9.3) | <.001 | 3,251 (9.0) | 1,910 (15.2) | <.001 |
| Maternal Education |  |  |  |  |  |  |  |  |  |
| <12 years | 2,760 (18.7) | 777 (36.1) | <.001 | 3,238 (10.9) | 1,969 (17.4) | <.001 | 3,255 (9.0) | 1,540 (12.2) | <.001 |
| 12 years | 4,789 (32.5) | 700 (32.5) | .992 | 7,065 (23.8) | 2,732 (24.1) | .488 | 7,011 (19.4) | 2,476 (19.7) | .573 |
| >12 years | 6,859 (46.5) | 512 (23.8) | <.001 | 18,510 (62.4) | 6,191 (54.7) | <.001 | 24,714 (68.4) | 8,144 (64.6) | <.001 |
| Medicaid Payment for Delivery | 7,270 (49.3) | 1,453 (67.4) | <.001 | 9,120 (30.7) | 4,738 (41.9) | <.001 | 9,175 (25.4) | 3,741 (29.7) | <.001 |
| Enrolled in WIC | 8,379 (56.8) | 1,481 (68.7) | <.001 | 10,875 (36.7) | 5,175 (45.7) | <.001 | 10,929 (30.3) | 4,239 (33.6) | <.001 |
| Race and Ethnicity |  |  |  |  |  |  |  |  |  |
| White, not Hispanic | 3,819 (25.9) | 439 (20.4) | <.001 | 10,583 (35.7) | 4,628 (40.9) | <.001 | 15,441 (42.8) | 5,295 (42.0) | .145 |
| Hispanic | 7,233 (49.0) | 829 (38.5) | <.001 | 10,767 (36.3) | 3,469 (30.7) | <.001 | 10,942 (30.3) | 3,517 (27.9) | <.001 |
| Black | 851 (5.8) | 346 (16.1) | <.001 | 1,279 (4.3) | 840 (7.4) | <.001 | 1,287 (3.6) | 641 (5.1) | <.001 |
| Asian | 1,745 (11.8) | 228 (10.6) | .091 | 4,869 (16.4) | 1,486 (13.1) | <.001 | 6,054 (16.8) | 2,080 (16.5) | .499 |
| Other | 1,100 (7.5) | 313 (14.5) | <.001 | 2,170 (7.3) | 894 (7.9) | .044 | 2,384 (6.6) | 1,069 (8.5) | <.001 |
| Body mass index |  |  |  |  |  |  |  |  |  |
| Underweight (<18.5 kg/m) | 743 (5.0) | 131 (6.1) | .042 | 1,708 (5.8) | 508 (4.5) | <.001 | 1,924 (5.3) | 753 (6.0) | .006 |
| Normal (18.5-24.9 kg/m^2^) | 7,281 (49.4) | 571 (26.5) | <.001 | 15,776 (53.2) | 6,438 (56.9) | <.001 | 21,319 (59.0) | 6,631 (52.6) | <.001 |
| Overweight (25.0-29.9 kg/m^2^) | 3,269 (22.2) | 487 (22.6) | .652 | 6,482 (21.9) | 1,858 (16.4) | <.001 | 6,824 (18.9) | 2,656 (21.1) | <.001 |
| Obese (≥30 kg/m^2^) | 2,626 (17.8) | 699 (32.4) | <.001 | 3,971 (13.4) | 1,827 (16.1) | <.001 | 4,068 (11.3) | 1,812 (14.4) | <.001 |
| Gestational diabetes | 697 (4.7) | 365 (16.9) | <.001 | 1,686 (5.7) | 863 (7.6) | <.001 | 1,832 (5.1) | 943 (7.5) | <.001 |
| Gestational hypertension | 483 (3.3) | 256 (11.9) | <.001 | 1,133 (3.8) | 644 (5.7) | <.001 | 1,261 (3.5) | 705 (5.6) | <.001 |
| Preeclampsia | 562 (3.8) | 350 (16.2) | <.001 | 1,017 (3.4) | 809 (7.2) | <.001 | 1,219 (3.4) | 804 (6.4) | <.001 |
| Smoking during pregnancy | 482 (3.3) | 459 (21.3) | <.001 | 779 (2.6) | 812 (7.2) | <.001 | 837 (2.3) | 674 (5.4) | <.001 |
| Drug or alcohol abuse | 115 (0.8) | 311 (14.4) | <.001 | 171 (0.6) | 530 (4.7) | <.001 | 182 (0.5) | 420 (3.3) | <.001 |
| Mental health diagnosis | 185 (1.3) | 387 (18.0) | <.001 | 422 (1.4) | 675 (6.0) | <.001 | 476 (1.3) | 593 (4.7) | <.001 |
| Previous preterm birth | 848 (5.8) | 614 (28.5) | <.001 | 1,624 (5.5) | 1,152 (10.2) | <.001 | 1,810 (5.0) | 1,133 (9.0) | <.001 |
| Outcomes |  |  |  |  |  |  |  |  |  |
| GA <37 weeks GA (preterm) | 1,186 (8.0) | 279 (13.0) | <.001 | 1,582 (5.3) | 754 (6.7) | <.001 | 1,597 (4.4) | 730 (5.8) | <.001 |
| GA 37-38 weeks GA (early term) | 4,401 (29.8) | 705 (32.7) | .007 | 8,216 (27.7) | 3,133 (27.7) | .985 | 9,359 (25.9) | 3,391 (26.9) | .030 |
| SGA | 1,024 (6.9) | 210 (9.7) | <.001 | 1,753 (5.9) | 741 (6.6) | .016 | 786 (6.2) | 1,949 (5.4) | <.001 |

GA: gestational age at birth, SGA: small for gestational age, WIC: Women, Infants, and Children Program

*Online Resource 3. Odds of Preterm Birth Subtype by Interpregnancy Interval in a Propensity Score- Matched Sample (n=83,788)*

| **Birth Outcome** |  |  |  |  | **Interpregnancy Interval Subgroups**  n (%)  OR (95% CI) | |  |  |  |
| --- | --- | --- | --- | --- | --- | --- | --- | --- | --- |
|  |  | <6 months  n=14,748 | 18-23 months  n=14,748 |  | 6-11 months  n=29,668 | 18-23 months  n=29,668 |  | 12-17 months  n=36,108 | 18-23 months  n=36,108 |
| GA <32 weeks GA |  | 131 (0.9) | 58 (0.4) |  | 155 (0.5) | 102 (0.3) |  | 142 (0.4) | 120 (0.3) |
|  |  | 2.50 (1.83, 3.41) | Ref |  | 1.58 (1.23, 2.03) | Ref |  | 1.19 (0.94, 1.52) | Ref |
| PPROM |  | 33 (0.2) | 23 (0.2) |  | 43 (0.1) | 34 (0.1) |  | 50 (0.1) | 39 (0.1) |
|  |  | 1.59 (0.93, 2.71) | Ref |  | 1.31 (0.84, 2.06) | Ref |  | 1.29 (0.85, 1.97) | Ref |
| Spontaneous |  | 89 (0.6) | 27 (0.2) |  | 93 (0.3) | 53 (0.2) |  | 79 (0.2) | 66 (0.2) |
|  |  | 3.65 (2.37, 5.62) | Ref |  | 1.82 (1.30, 2.55) | Ref |  | 1.21 (0.87, 1.67) | Ref |
| Indicated |  | 4 (0.0) | 5 (0.0) |  | 13 (0.0) | 10 (0.0) |  | 10 (0.0) | 9 (0.0) |
|  |  | 0.89 (0.24, 3.30) | Ref |  | 1.35 (0.59, 3.08) | Ref |  | 1.12 (0.46, 2.76) | Ref |
| GA 32-36 weeks GA |  | 1,055 (7.2) | 637 (4.3) |  | 1,427 (4.8) | 1,247 (4.2) |  | 1,455 (4.0) | 1,474 (4.1) |
|  |  | 1.83 (1.66, 2.03) | Ref |  | 1.19 (1.10, 1.29) | Ref |  | 1.00 (0.92, 1.07) | Ref |
| PPROM |  | 187 (1.3) | 137 (0.9) |  | 265 (0.9) | 285 (1.0) |  | 294 (0.8) | 349 (1.0) |
|  |  | 1.51 (1.21, 1.89) | Ref |  | 0.97 (0.82, 1.14) | Ref |  | 0.85 (0.73, 0.99) | Ref |
| Spontaneous |  | 625 (4.2) | 325 (2.2) |  | 810 (2.7) | 645 (2.2) |  | 756 (2.1) | 756 (2.1) |
|  |  | 2.13 (1.86, 2.44) | Ref |  | 1.30 (1.17, 1.45) | Ref |  | 1.00 (0.91, 1.12) | Ref |
| Indicated |  | 157 (1.1) | 125 (0.9) |  | 236 (0.8) | 227 (0.8) |  | 288 (0.8) | 263 (0.7) |
|  |  | 1.39 (1.10, 1.76) | Ref |  | 1.08 (0.90, 1.30) | Ref |  | 1.10 (0.93, 1.31) | Ref |
| GA <37 weeks GA (any preterm) |  | 1,186 (8.0) | 695 (4.7) |  | 1,582 (5.3) | 1,349 (4.6) |  | 1,597 (4.4) | 1,594 (4.4) |
|  |  | 1.89 (1.71, 2.08) | Ref |  | 1.22 (1.13, 1.31) | Ref |  | 1.01 (0.94, 1.09) | Ref |
| PPROM |  | 220 (1.5) | 160 (1.1) |  | 308 (1.0) | 319 (1.1) |  | 344 (1.0) | 388 (1.1) |
|  |  | 1.52 (1.24, 1.87) | Ref |  | 1.00 (0.86, 1.17) | Ref |  | 0.89 (0.77, 1.04) | Ref |
| Spontaneous |  | 714 (4.8) | 352 (2.4) |  | 903 (3.0) | 698 (2.4) |  | 835 (2.3) | 822 (2.3) |
|  |  | 2.25 (1.97, 2.56) | Ref |  | 1.34 (1.21, 1.49) | Ref |  | 1.02 (0.93, 1.13) | Ref |
| Indicated |  | 161 (1.1) | 130 (0.9) |  | 249 (0.8) | 237 (0.8) |  | 298 (0.8) | 272 (0.8) |
|  |  | 1.37 (1.09, 1.73) | Ref |  | 1.09 (0.91, 1.30) | Ref |  | 1.11 (0.94, 1.30) | Ref |

GA: gestational age at birth, OR: odds ratio, PPROM: preterm prelabor rupture of the membranes; ^a^ *p*<.05
